# Prediction of new onset postoperative atrial fibrillation using a simple Nomogram

**DOI:** 10.1186/s13019-023-02198-1

**Published:** 2023-04-12

**Authors:** Siming Zhu, Hebin Che, Yunlong Fan, Shengli Jiang

**Affiliations:** 1 Medical School of Chinese PLA, Beijing, 100853 China; 2 Department of Cardiovascular Surgery, The First Medical Center of Chinese PLA General Hospital, No. 28 Fuxing Rd, Beijing, 100853 China

**Keywords:** Cardiac surgery, Surgical complications, Atrial fibrillation, Risk stratification

## Abstract

**Background:**

New onset postoperative atrial fibrillation (POAF) is the most common complication of cardiac surgery, with an incidence ranging from 15 to 50%. This study aimed to develop a new nomogram to predict POAF using preoperative and intraoperative risk factors.

**Methods:**

We retrospectively analyzed the data of 2108 consecutive adult patients (> 18 years old) who underwent cardiac surgery at our medical institution. The types of surgery included isolated coronary artery bypass grafting, valve surgery, combined valve and coronary artery bypass grafting (CABG), or aortic surgery. Logistic regression or machine learning methods were applied to predict POAF incidence from a subset of 123 parameters. We also developed a simple nomogram based on the strength of the results and compared its predictive ability with that of the CHA2DS2-VASc and POAF scores currently used in clinical practice.

**Results:**

POAF was observed in 414 hospitalized patients. Logistic regression provided the highest area under the receiver operating characteristic curve (ROC) in the validation cohort. A simple bedside tool comprising three variables (age, left atrial diameter, and surgery type) was established, which had a discriminative ability with a ROC of 0.726 (95% CI 0.693–0.759) and 0.727 (95% CI 0.676–0.778) in derivation and validation subsets respectively. The calibration curve of the new model was relatively well-fit (*p* = 0.502).

**Conclusions:**

Logistic regression performed better than machine learning in predicting POAF. We developed a nomogram that may assist clinicians in identifying individuals who are prone to POAF.

## Introduction

New-onset postoperative atrial fibrillation (POAF) is the most common complication after adult cardiac surgery, with a reported incidence rate from 15 to 50% [1]. New-onset atrial fibrillation, including paroxysmal, persistent, or permanent after any heart surgery, may be classified as POAF. It has been found that POAF after cardiac surgery is associated with a substantial risk of adverse outcomes, including increased mortality, postoperative stroke, respiratory infections, and gastrointestinal dysfunction [2]_._ Additionally, POAF increases hospitalization length and costs [3]_._ In contrast, POAF prophylaxis can cause hypotension, bradycardia, or heart block. Identifying patients prone to POAF makes personalized prophylactic treatment feasible. Several predictive models have been developed to achieve this goal. Our study aimed to develop a simple yet valid risk assessment model to predict POAF after cardiac surgery. Machine learning and logistic regression algorithms were used to develop a predictive model. To our knowledge, this is the first study to compare different algorithms for developing a POAF risk model.

## Methods

### Study population

This retrospective study included 2108 consecutive adult patients (age ≥ 18 years) who underwent cardiac surgery at the First Medical Center of Chinese PLA General Hospital (Beijing, China) from January 2018 to December 2020. The surgery types included isolated coronary artery bypass grafting (CABG), valve surgery, concomitant valve and CABG, and valve and ascending aorta surgery. Elective, urgent, and emergency procedures were also performed. The exclusion criteria were: age < 18 years, incomplete or non-availability of medical records, non-sinus electrocardiogram before surgery, medical history of atrial fibrillation or atrial flutter, pacemaker implantation, history of radiofrequency ablation or Cox maze procedure for arrhythmia, and death during the perioperative period. The final sample consisted of 1587 patients. The study was approved by the institutional review board of the Chinese PLA General Hospital (approval number S2022-360-01). Given the observational nature of this study, the requirement for informed consent was waived. All identifiable information about the patients was hidden, and their identities could not be determined based on context. This study was conducted following the Declaration of Helsinki and its amendments.

### Study endpoint

The primary outcome was the occurrence of new-onset POAF, as a binary value. POAF was defined as any episode of atrial fibrillation (AF) (occurrence of irregular heart rhythm, without detectable P waves) lasting more than 30 s on cardiac telemetry or requiring treatment (including antiarrhythmic drugs, such as amiodarone or electrical cardioversion) during hospitalization. This standard is consistent with most previous studies [2]_._ All patients underwent continuous ECG monitoring in the ICU for at least 48 h postoperatively. After the telemetry was removed, a standard 12-lead ECG was routinely recorded on the first and third day after leaving the ICU. Even without telemetry, episodes of AF were detected by a change in clinical status, which led to an immediate bedside electrocardiogram. ECG and telemetry were double-checked by a cardiac surgeon and an electrophysiologist. If there was a disagreement, a third cardiologist was required to judge the ECGs.

### Data collection and statistical analysis

All clinical data were extracted from the electronic medical records of our hospital. Two separate investigators independently collected the data and proofread each other's collections. A total of 123 preoperative and intraoperative characteristics were collected from all patients, including demographic data, medical history, preoperative medications, preoperative laboratory tests, preoperative echocardiography, electrocardiography, and intraoperative variables. All collected data were preprocessed and cleaned before analysis, and no extreme or missing values were found. The normality of continuous variables in baseline characteristics was assessed using the Shapiro–Wilk test. Normally distributed continuous data were estimated by Student's t-test and demonstrated as mean ± standard deviation (SD). In the case of non-normally distributed variables, Mann–Whitney U tests were used for comparison and are presented as median (IRQ, 25–75th percentiles). Categorical variables were presented as percentages and compared using the Pearson chi-square test. Univariate analysis was used to select variables for further inclusion in the multivariate logistic regression models based on *p*-values of less than 0.1. Multivariate analysis was performed using stepwise logistic regression with backward selection, and two-sided *p* values < 0.05 were considered an statistically significant. Comparisons between the areas under the receiver operating characteristic (ROC) curves were performed using DeLong’s test. The Hosmer–Lemeshow test was used to calibrate the model. Clinical data were recorded and tabulated using Microsoft Excel, and all analyses were performed using R software, version 4.1.1.

### Model development

The data collected were randomly divided into derivation subset and validation subsets. The derivation subset contained 70% of patients and was used for training the model, whereas the validation subset contained the remaining 30% of patients and was used for model testing. We applied logistic regression and three machine learning methods that are popular in dealing with binary problems: random forests (Forest), Naïve Bayes (Bayes), and k-Nearest Neighbor (KNN). Univariate analysis was performed to select variables based on *p-*values of less than 0.1. Our study examined whether the three machine learning methods should use all 123 parameters or only those selected by univariate analysis to select the best scenario. For the logistic regression (Logistic) model, only data selected by univariate analysis were included in the multivariable analysis. We also attempted a ten-fold cross-validation process in which the derivation subset was randomly divided into ten almost equal groups, called folds. In every test, model training was performed on a smaller subset consisting of nine folds, and then the model’s performance was evaluated using the withheld fold. This procedure was performed for all ten folds, therefore, we repeated this process ten times to obtain ten individual probabilities. The final performance for each model was the mean of the ten individual probabilities. The models built from the derivation subset were fed into the validation subset for validation. A validation subset was used to compare the performance of each model, and the one with the best performance was chosen to construct the nomogram.

## Results

### Characteristics of patients

**Fig. 1:**
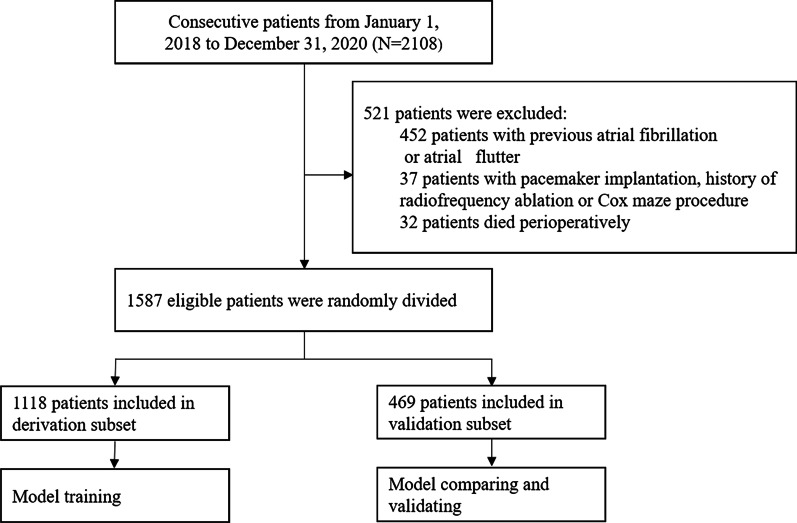
The flow chart displays the inclusion and exclusion of patients

A total of 2108 consecutive adult patients underwent cardiac surgery between January 1, 2018, and December 31, 2020, of whom 1587 were enrolled (Fig. 1). The basic demographic and surgical characteristics of patients are summarized in Table 1. The median age was 58.0 years (49.0–66.0), 517 (32.6%) were women, 1070 (67.4%) were men, and 634 (39.9%) patients had a history of smoking. The percentage of patients with hypertension, COPD, diabetes, and renal dysfunction was 47.9%, 5.9%, 20.9%, and 3.8%, respectively. Valve surgery accounted for 45.3% of cases, CABG for 36.3%, and concomitant valve and CABG surgery or aortic surgery for the rest, among which 5.7% were emergent cases. The median operation duration was 280.0 min (235.0–340), and the median cardiopulmonary bypass time (CPB) time was 116.0 min (85.0–161.0).

**Table 1:** Demographical and surgical characteristics of all patients

Variables	Overall (n = 1587)
Demographic data	
Age, years, median (IQR)	58.0 (49.0–66.0)
Sex, male, n (%)	1070 (67.4%)
Body-mass index, kg/m^2^ median (IQR)	25.2 (22.7–27.4)
Smoking, n (%)	634 (39.9%)
NYHA class I/II/III/IV, n (%)	439(27.7%)/654(41.2%)/442(27.9%)/52(3.3%)
ASA class I/II/III/IV/V, n (%)	8(0.5%)/292(18.4%)/837(52.7%)/448(28.2%)/ 2(0.1%)
EuroSCORE II, median (IQR)	1.3 (0.8–2.8)
CHA2DS2-VASc score, median (IQR)	2.0 (1.0–3.0)
Medical history, n (%)	
Hypertension	760 (47.9%)
COPD	94 (5.9%)
Previous MI	84 (5.3%)
Previous stroke	86 (5.4%)
Diabetes	332 (20.9%)
CKD	61 (3.8%)
eGFR, ml/min/1.73 m^2^, median (IQR)	84.1 (67.5–102.7)
Surgery characteristics	
Surgery type, n (%)	
Valve	719 (45.3%)
CABG	576 (36.3%)
Valve + CABG	128 (8.1%)
Valve + aorta	164 (10.3%)
Emergency, n (%)	91 (5.7%)
CPB time, min, median (IQR)	116.0 (85.0–161.0)
Clamping time, min, median (IQR)	88.0 (64.0–124.0)
Operation duration, min, median (IQR)	280.0 (235.0–340)

*ASA* American Society of Anesthesiologists Physical Status, *CKD* Chronic kidney disease, *COPD* chronic obstructive pulmonary disease, *CPB* cardiopulmonary bypass, *eFGR* estimated glomerular filtration rate, *IQR* interquartile range, *MI* myocardial infarction, *NYHA* New York Heart Association

### Derivation and validation subsets

The randomly assigned derivation and validation subsets enrolled 1118 (70.4%) and 469 (29.6%) patients, respectively. No significant differences were observed in variables between the derivation and validation subsets. There were 293 and 121 patients who developed POAF in the derivation and validation subsets, respectively (26.2% vs 25.8%, *p* = 0.792). Results of the univariate analysis of parameters associated with POAF in the derivation are reported in Table 2 (*p* < 0.1). Patients who developed POAF were older, heavier, and had higher ASA (American Society of Anesthesiologists Physical Status) IV/V and NYHA (New York Heart Association) III/IV percentages. They also exhibited larger left atrial diameter (LAd), interventricular septum (IVS), and left ventricular posterior wall (LVPW) but lower left ventricular ejection fraction (LVEF) on echocardiography. Among the laboratory parameters, lower red blood cell (RBC) count, platelet count, total plasma protein, plasma albumin, and estimated glomerular filtration rate (eGFR) were associated with an increased frequency of POAF. Patients who underwent valvular surgery, concomitant CABG and valvular surgery were more likely to develop POAF. Intraoperative medication usage demonstrated no significant difference, except for inotropic drugs such as epinephrine, norepinephrine, and isoproterenol, which were associated with POAF. Univariate analysis also suggested that surgical characteristics, such as blood loss volume, packed red blood cell (pRBC) transfusion volume, fresh frozen plasma (FFP) transfusion volume, CPB, and anesthesia time were associated with POAF.

**Table 2:** Univariable analyses of the patients in the derivation cohort

Variables	No POAF(n = 825)	POAF (n = 293)	*P* valve
Age, years, median (IQR)	56.0 (45.0–65.0)	63 (55.0–69.0)	< 0.001
Weight, kg, median (IQR)	69.5 (60.0–78.0)	67.0 (58.8–75.9)	0.083
ASA, n (%)			0.002
I	6 (0.7%)	0 (0.0%)	
II	174 (21.1%)	38 (13.0%)	
III	440 (53.3%)	159 (54.3%)	
IV	204 (24.7%)	95 (32.4%)	
V	1 (0.1%)	1 (0.34)	
NYHA			0.015
I	241 (29.2%)	69 (23.5%)	
II	342 (41.5%)	123 (42.0%)	
III	224 (27.2%)	85 (29.0%)	
IV	18 (2.2%)	16 (5.5%)	
EuroSCORE II, median (IQR)	1.18 (0.75–2.35)	1.83 (0.93–3.70)	< 0.001
Medical history, n (%)			
Preoperative Diuretics	532 (64.5%)	218 (74.4%)	0.002
CKD	20 (2.4%)	15 (5.1%)	0.037
Preoperative laboratory values			
RBC, × 10^9^/L, median (IQR)	4.45 (4.07–4.82)	4.28 (3.88–4.75)	< 0.001
Hgb, g/dL, median (IQR)	136 (121–147)	131 (118–145)	0.020
HCT, %, median (IQR)	0.39 (0.36–0.43)	0.38 (0.35–0.42)	0.012
Platelet, × 10^9^/L, median (IQR)	202 (166–245)	185 (150–229)	< 0.001
Total plasma protein, g/L, median	67.7 (64.0–71.8)	66.6 (63.5–70.8)	0.019
Plasma albumin, g/L, median, (IQR)	41.2 (38.5–43.9)	40.4 (37.4–43.0)	0.001
Scr, μmoI/L, median, (IQR)	77.9 (66.7–89.3)	80.1 (68.6–92.5)	0.031
Blood urea nitrogen, mmol/L, median	5.7 (4.5–6.7)	6.3 (5.0–7.5)	< 0.001
eGFR, ml/min/1.73 m2, median (IQR)	88.0 (71.0–106.0)	74.8 (61.5–91.7)	< 0.001
Preoperative echocardiography			
LAd, mm, median (IQR)	37.0 (33.0–42.0)	40.0 (35.0–46.0)	< 0.001
IVS, mm, median (IQR)	11.0 (10.0–12.0)	12.0 (10.0–13.0)	0.002
LVPW, mm, median (IQR)	11.0 (10.0–12.0)	11.0 (10.0–13.0)	0.002
LVEF, %, median (IQR)	61.0 (56.0–68.0)	60.0 (52.0–66.0)	0.008
Surgery characteristics, n (%)			
Surgery type			< 0.001
Valve	389 (47.2%)	136 (46.4%)	
CABG	307 (37.2%)	90 (30.7%)	
Valve + CABG	42 (5.1%)	36 (12.3%)	
Valve + aorta	87 (10.5%)	31 (10.6%)	
MIS, n (%)	183 (22.2%)	47 (16.0%)	0.032
Inotropic drug, n (%)	57 (0.48%)	44 (15%)	< 0.001
PLT transfusion	197 (23.9%)	102 (34.8%)	0.001
FFP transfusion, unit, median (IQR)	4.2 (0.0–5.2)	4.9 (0.0–5.9)	< 0.001
pRBC transfusion, unit, median (IQR)	2.0 (0.0–4.0)	2.5 (0.0–4.0)	< 0.001
Blood loss, ml, median (IQR)	300 (300–400)	400 (300–500)	< 0.001
Urine output, ml, median (IQR)	870 (500–1300)	800 (400–1290)	0.045
CPB time, min, median (IQR)	114 (84–157)	125 (92–170)	0.017
Anesthesia time, min, median (IQR)	325 (280–390)	340 (290–405)	0.093
Operation time, min, median (IQR)	270 (230–340)	285 (240–350)	0.077

*FFP* fresh frozen plasma, *HCT* hematocrit, *Hgb* hemoglobin, *MIS* minimally invasive surgery, *PLT* platelet, *Scr* serum creatinine

### Model discrimination

**Fig. 2:**
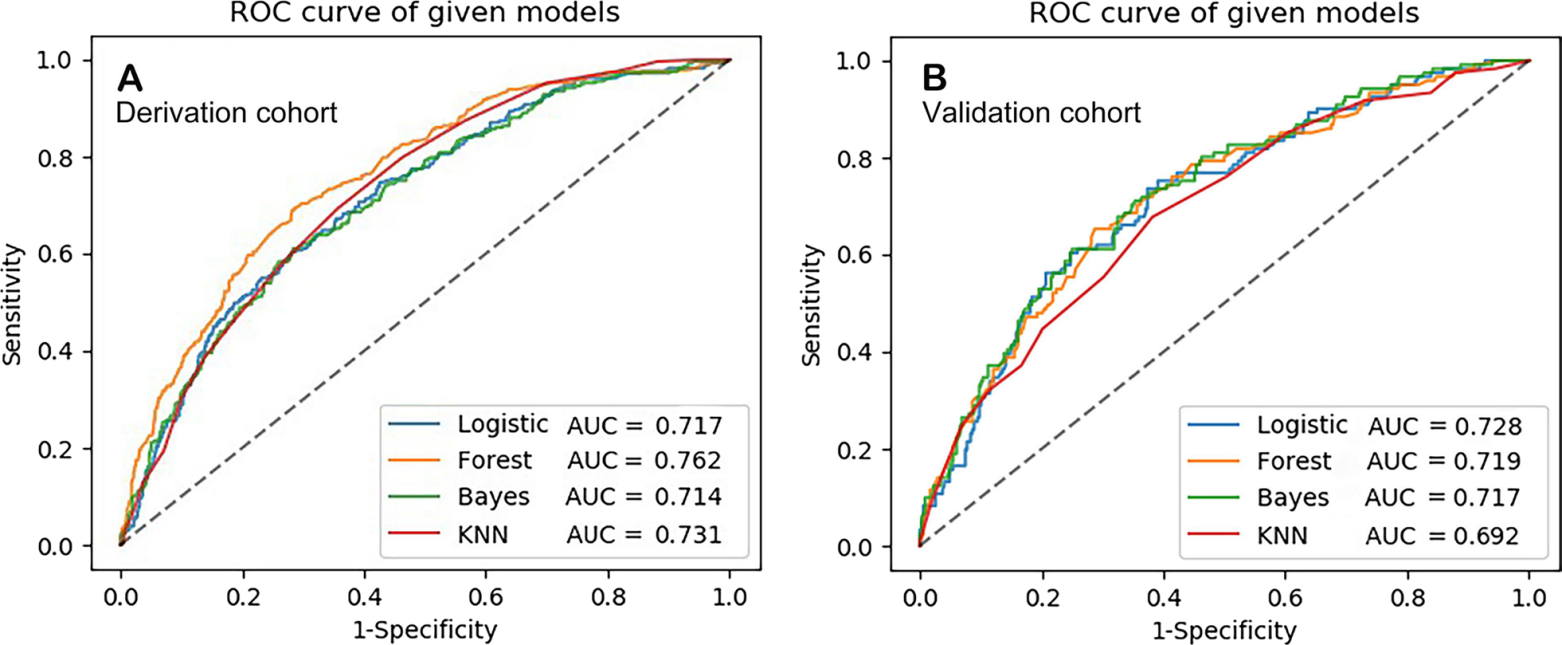
Area under the ROC curve showing the performance of different models in predicting POAF in derivation (**A**) and validation (**B**) cohorts

Figure 2 depicts the performance of the different models. In the derivation cohort, the Forest model had an area under the ROC curve of 0.762 (95% CI 0.730–0.793), followed by the second was the KNN model (ROC: 0.731, 95% CI 0.699–0.762). The logistic model yielded a ROC of 0.717 (95% CI 0.683–0.750) in the training cohort, which only ranked higher than that of the Bayes model (ROC: 0.714, 95% CI 0.680–0.762), exhibiting the best performance (ROC: 0.728, 95% CI 0.673–0.767–0.779) in the validation cohort. Delong’s test indicated that all four models had relatively equal discrimination performance in the two cohorts (all *p* > 0.05). Notably, compared with the derivation cohort, both the Forest and KNN models exhibited a decline in predictive ability in the validation cohort, whereas the logistic model demonstrated the opposite.

### Development and calibration of the nomogram

**Fig. 3:**
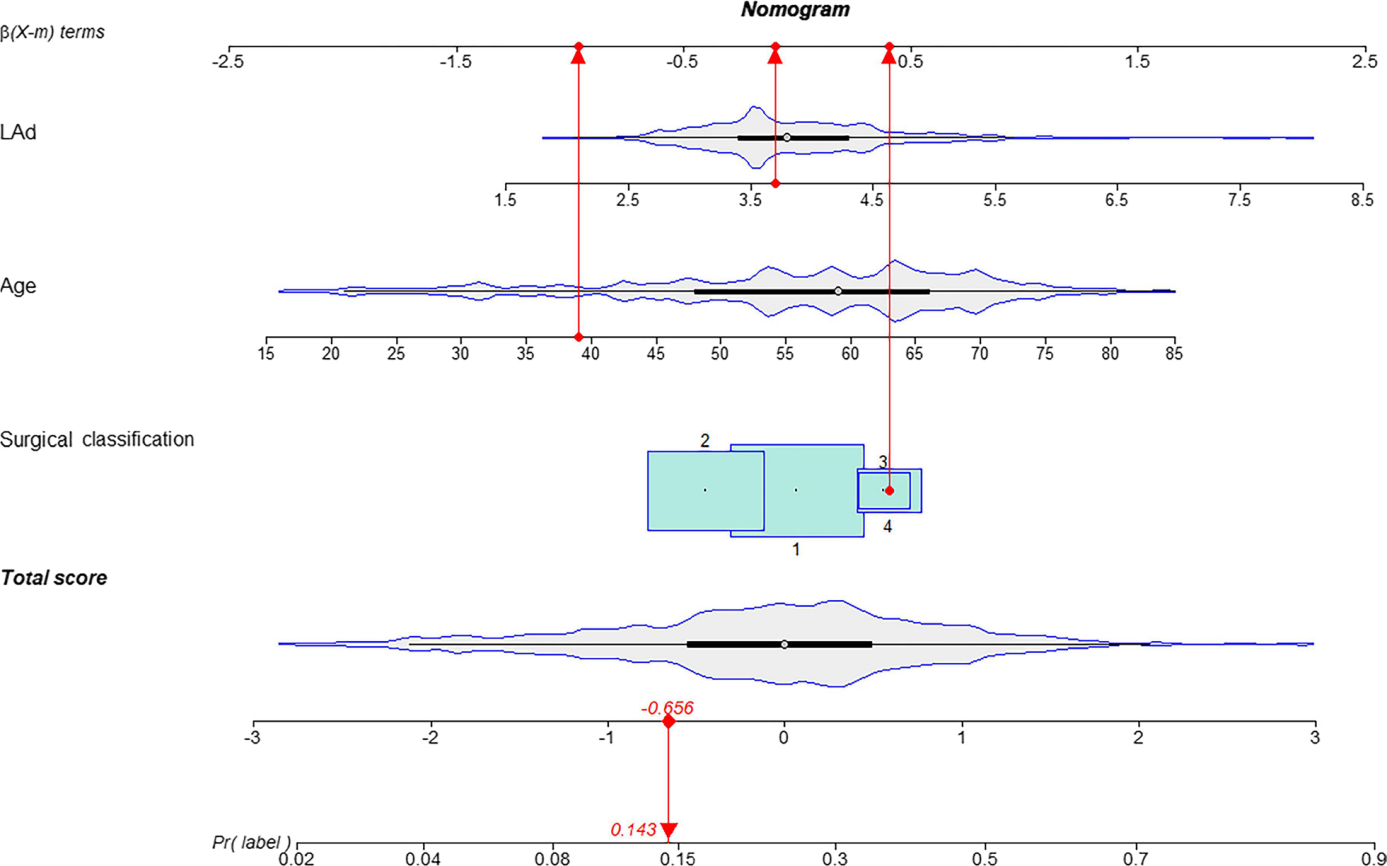
A constructed nomogram for predicting POAF. For surgical classification variables, their distributions are reflected by the box size (boxes 1 to 4 represent valve surgery, CABG, concomitant valve and CABG surgery, and aorta surgery, respectively). *LAd* left atrial diameter, *Pr* probability

**Fig. 4:**
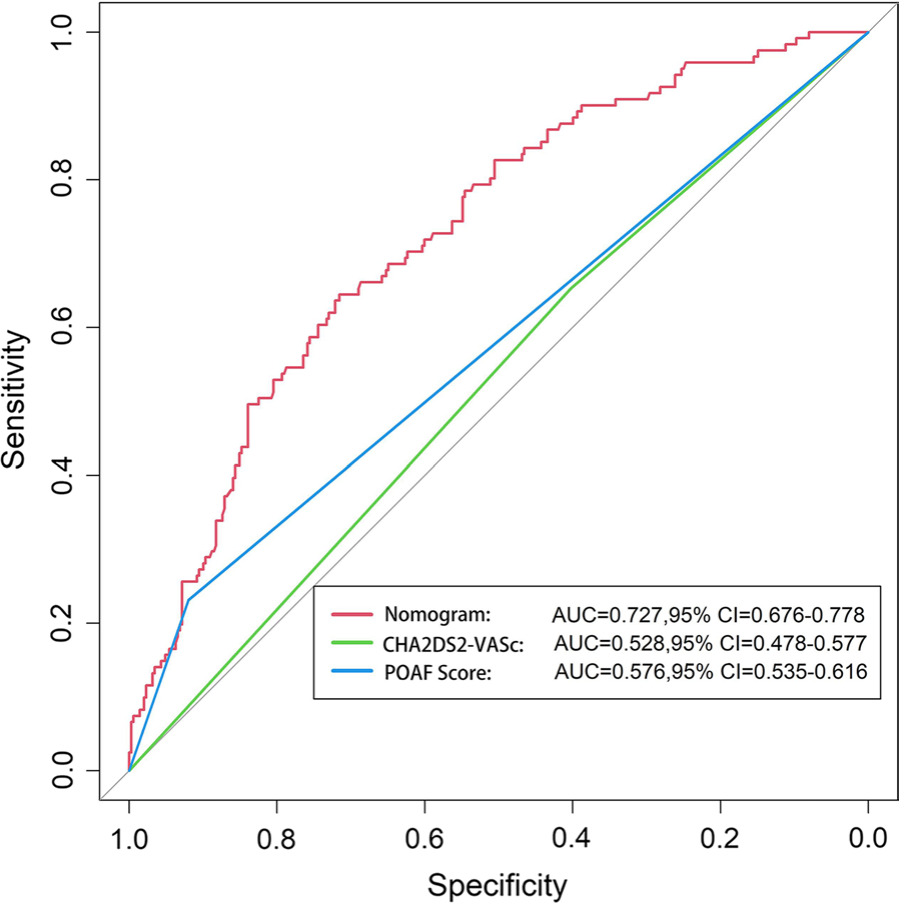
Area under the ROC curve depicts the performance of the nomogram, POAF score, and CHA2DS2-VASc score. *CI* confidence interval

**Fig. 5:**
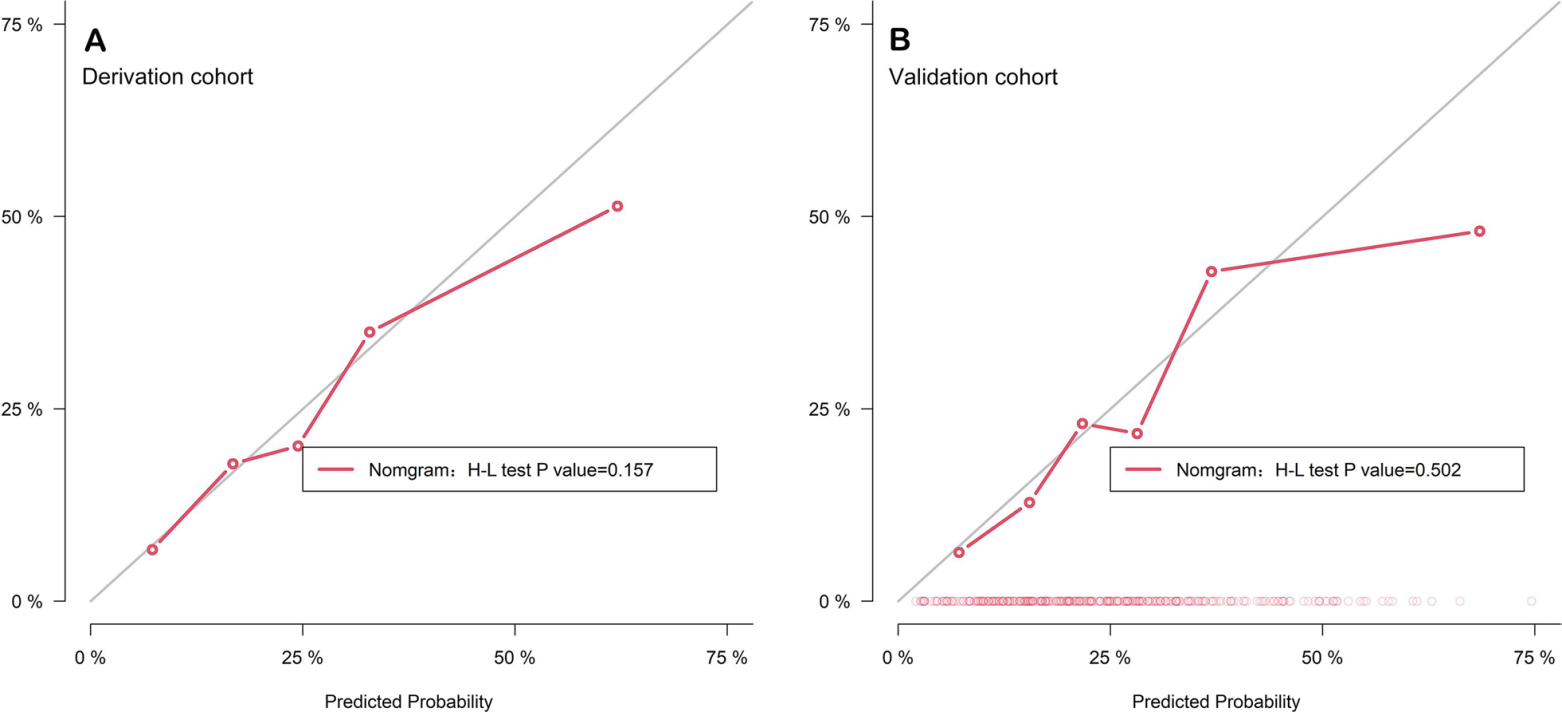
Calibration curve of the model in the derivation (**A**) and validation (**B**) cohort. The Y-axis represents the actual POAF incidence rate, and the x-axis represents the predicted rate. The diagonal line represents a perfect prediction by the ideal model. The red line represents the performance of the nomogram, of which a closer fit to the diagonal line represents a better prediction

Considering the performance of each model, we developed a nomogram based on the logistic model. The important independent predictive factors identified by multivariate analysis were age (OR, 1.06; 95% CI 1.04–1.07; *p* < 0.001), LAd (OR, 1.71; 95% CI 1.41–2.10; *p* < 0.001), surgery type (CABG: OR, 0.67; 95% CI 0.46–0.96. Valve + CABG: OR, 1.46; 95% CI 0.87–2.46. Valve + aorta: OR, 1.50; 95% CI 0.90–2.46, *p* = 0.03). In the nomogram, all these variables were assigned a score on a point scale so that the total score could be achieved by adding them. To use the nomogram, the specific points (red dots) of individual patients are located on each variable axis. Figure 3 displays the prediction of POAF occurrence in a 39-year-old patient with a left atrial diameter of 3.8 cm who underwent aortic valve and ascending aorta replacement. Red lines and dots are drawn upward to determine the points received by each variable; the sum of these points (-0.656) is located on the Total Score axis, and a line is drawn downward to the lowest axes (probability scale) to determine the probability of POAF (14.3%) (Fig. 3). The area under the ROC curve of the nomogram was 0.726 (95% CI 0.693–0.759) and 0.727 (95% CI 0.676–0.778) in the training and testing cohorts, respectively. In comparison, the ROC of POAF and CHA2DS2-VASc scores were 0.576 (95% CI 0.535–0.616) and 0.528 (95% CI 0.478–0.577, Fig. 4). The calibration curve revealed that the nomogram had fair agreement between the observed and expected rates of POAF in both the derivation (*p* = 0.157) and validation cohorts (*p* = 0.502, Fig. 5).

## Discussion

Postoperative atrial fibrillation (POAF) is the most common complication after adult cardiac surgery, with approximately 25% of patients undergoing coronary artery bypass grafting (CABG), 30% undergoing isolated valvular surgery, and 40%–50% undergoing combined valve and CABG procedure [4]_._ Our study demonstrated an overall POAF rate of 26.1%, which was slightly lower than that reported in most previous studies [5], given that valve or concomitant valvular surgery accounted for 63.7% of all cases. Our study cohort was younger age and had a lower comorbidity rate, which may explain this difference.

Although some studies suggest that POAF is transient and benign after cardiac surgery, a growing number of studies indicate that it may be related to a higher mortality rate accompanied by numerous complications, including stroke, congestive heart failure, gastrointestinal dysfunction, and an eight-fold possibility of subsequent atrial fibrillation, as well as ICU stay time and costs [6, 7]_._ As a result, reducing POAF incidence may benefit patients medically and financially. Recent advances in equipment and improved surgical techniques have significantly reduced cardiac surgery-associated morbidity and mortality, however, the incidence of POAF remains relatively unchanged [5].

It is of great interest to clinicians to stratify patients based on their predisposition to POAF. Identifying high-risk patients and prophylactic treatment are more cost-effective than implementing preventive methods for all patients. This also minimizes prophylaxis caused by the side effects such as unstable hemodynamics, bradycardia, or heart arrest. Distinct preoperative risk models have been developed to achieve this goal. Among them, the most widely used models are the POAF and CHA_2_DS_2_-VASc scores which are well-known for their simplicity and ability to predict POAF.

The POAF score had a relatively better performance than the CHA2DS2-VASc score in our cohort, with an area under the receiver operating characteristic (ROC) curve of 0.576 (95% CI 0.535–0.616) and 0.528 (95% CI 0.478–0.577), respectively, which is consistent with most previous studies [8, 9]. Utilizing CHA2DS2-VASc or POAF scores to identify patients prone to developing POAF makes it possible to adopt personalized preventative strategies instead of providing all patients with prophylactic treatment. However, the observed performance is not very promising. Some of these reasons may be related to the two risk models. The POAF score was derived from a population with non-cardiac thoracic surgery, which may be the primary reason for poor performance in the cardiac surgery population. The CHA2DS2-VASc score was originally developed to guide antithrombotic treatment in patients with AF or atrial flutter and was subsequently found to help predict POAF after cardiac surgery. Intraoperative data were not included in determining the CHA2DS2-VASc score [10]. Other important variables, such as parameters of preoperative echocardiography, electrocardiogram, and intraoperative drugs, were also excluded in the process of developing CHA2DS2-VASc and POAF scores [9, 10]_._ These preoperative and intraoperative characteristics have been widely validated worldwide to increase POAF susceptibility [11–13]_._ Furthermore, these two predictive models were originally designed and developed in Europe, where participants were primarily Caucasian and possibly had a risk factor for POAF. A growing body of evidence indicates that ethnicity and genetic factors are related to POAF [1, 14, 15]_._ Even Asians, Chinese, and Malay Indians may be at a higher risk than Indians [16]. Therefore, the differences in patient characteristics and basic cardiac conditions from the original studies might explain the low performance of CHA2DS2-VASc and POAF scores.

Although POAF is not a unique complication of cardiac surgery, its incidence rate is significantly higher than that of non-cardiac surgery (1%–15%). Several studies have depicted that the pathophysiology of POAF varies between the types of surgery [17]_._ The mechanism of POAF after cardiac surgery has not yet been fully elucidated. The reported predisposing factors may include but are not limited to age, sex, left atrial diameter (LAd), blood pressure, and chronic obstructive pulmonary disease (COPD). However, a few parameters demonstrated statistical differences in univariable analysis. In contrast, multivariate analysisidentified age, LAd, and surgical type as significant independent predictors in our study. The nomogram built on the strength of these results demonstrated discriminative ability with ROC of 0.727 (95% CI 0.676–0.778) in the validation subset. The calibration test maintained an increasing incidence of POAF with an increasing score, calibrating best in patients at low risk but overpredicting the incidence with higher scores.

It is generally agreed that there are multiple mechanisms in POAF development and that age is the most recognized risk factor and predictor [18, 19]_._ The reasons for POAF incidence correlated with aging are diverse. Aging leads to myocardial fibrosis and collagen deposition in the atrium, rendering the atria more vulnerable to surgical conditions such as inflammation, hypovolemia, ischemia, and activated autonomic nervous system. Fibrosis and degeneration in the atrium weaken the coupling structure between myocardial fibers, resulting in slowed electrical conduction. Due to the different electrical properties of fibrotic and normal cardiomyocytes, this change can predispose to abnormal conduction and reentry of arrythmias [20].

Preoperative echocardiography is also a series of consistent risk indicators across multiple studies, among which left atrial dilation is the most recognized. Although there is heterogeneity in different studies on how LAd is defined, a left atrium diameter of more than 40 mm is mostly adopted. LAd may result from a reconstruction of atria due to pressure or volume overload caused by pathophysiological conditions such as mitral valve regurgitation, hypertension, and congestive heart failure. Left atrial reconstruction is also a manifestation of myocardial fibrosis [20]_._ A 2020 meta-analysis demonstrated that POAF patients had a larger mean LAd of 2.01 (95% CI 1.03–2.99) mm compared to patients without POAF [21]_._

Substantial evidence suggests that valvular surgery is associated with a greater incidence of POAF than isolated CABG. Some studies have demonstrated that POAF is most common after mitral valve interventions [22]_,_ whereas others have revealed that aortic surgery has the highest POAF rate [23]_._ However, patients undergoing valve or combined valvular surgery usually experience increased surgical trauma and longer time under cardiopulmonary bypass (CPB), which leads to increased sympathetic tone. Activated sympathetic nerves shorten the effective refractory period of atrial myocytes associated with POAF. This is supported by the fact that drugs with sympathetic activity enhance POAF. In contrast, β-blocking agents that can decrease sympathetic tone are significantly reduced.

Machine learning (ML) algorithms have gained popularity in medicine as a branch of artificial intelligence. The definition of ML and its differences from statistical models have not yet been defined [24]_._ In this study, we adopted the classification that ML is data orientated, whereas traditional algorithms such as logistic regression (LR) are based on theory and assumptions. Some studies demonstrated that ML could be superior to LR in building prediction models because ML had better performance when dealing with a large number of potential predictors and did not require independent or nonlinear [25, 26]_,_ the outcomes of some studies were found to be otherwise [27]_._

In our study, LR depicted the second-best ROC in the derivation cohort and achieved the best ROC in the validation cohort. A main drawback of ML is the elevated risk of overfitting, which may result from down sampling or insufficient sample size. In this study, LG exhibited a better performance in the validation cohort than in the derivation cohort, which contrasts with the performance of all ML models. Based on accuracy, transparency, and interpretability, we selected LR to build the prediction model.

## Study limitations

The limitations of our study are that patients in the derivation cohort may not be sufficient for ML algorithms, which tend to yield better performance with increasing cases. The second limitation was the inherent limitations of this retrospective study. Third, this was a single-center study. The nomogram requires a larger, multicenter sample for external validation. Finally, the patients did not receive continuous electrocardiographic monitoring until discharge, therefore, episodes of asymptomatic POAF may have been missed, leading to an underestimation of the POAF incidence.

## Conclusions

We constructed a nomogram to identify patients at risk of developing POAF after cardiac surgery to take prophylactic measures more efficiently. This will likely yield better discriminatory power than the widely used POAF and CHA2DS2-VASc scores. However, the nomogram requires further validation using larger, multicenter samples.

## Data Availability

The datasets used and/or analyzed during the current study are available from the corresponding author upon reasonable request.

## Abbreviations

AF: Atrial fibrillation
ASA: American Society of Anesthesiologists Physical Status
CABG: Coronary artery bypass graft
CI: Confidence interval
CKD: Chronic kidney disease
COPD: Chronic obstructive pulmonary disease
CPB: Cardiopulmonary bypass
ECG: Electrocardiogram
eFGR: Estimated glomerular filtration rate
FFP: Fresh freezing plasma
HCT: Hematocrit
Hgb: Hemoglobin
ICU: Intensive care unit
IRQ: Interquartile range
IVS: Interventricular septum
KNN: K-Nearest Neighbor
LAd: Left atrial diameter
LVEF: Left ventricular ejection fraction
LVPW: Left ventricular posterior wall
MI: Myocardial infarction
MIS: Minimally invasive surgery
NYHA: New York Heart Association
PLT: Platelet
POAF: New onset postoperative atrial fibrillation
Pr: Probability
ROC: The receiver operating characteristic curves
SD: Standard deviation
Scr: Serum creatinine
